# Supplementary material for: Integration of microbattery with thin-film electronics for constructing an integrated transparent microsystem based on InGaZnO
Source: Nat Commun. 2023 Sep 1;14:5330. doi: 10.1038/s41467-023-41181-1 (PMC10474284; doi:10.1038/s41467-023-41181-1)
Supplement: Supplementary file 1 — Supplementary Information [file 41467_2023_41181_MOESM1_ESM.pdf]

Supplementary Information

Integration of microbattery with thin-film electronics for constructing an integrated transparent microsystem based on InGaZnO

Bin Jia[1], Chao Zhang[1], Min Liu[1], Zhen Li[1], Jian Wang[1], Li Zhong[1], Chuanyu Han[2], Ming Qin[1], Xiaodong Huang[1]*

[1]Key Laboratory of MEMS of the Ministry of Education, School of Integrated Circuit, Southeast University, Nanjing, 210096, China

[2]School of Microelectronics, Faculty of Electronics and Information, Xi'an Jiaotong University, Xi'an, 710049, China

* Corresponding author

E-mail address: xdhuang@seu.edu.cn (X.D. Huang)

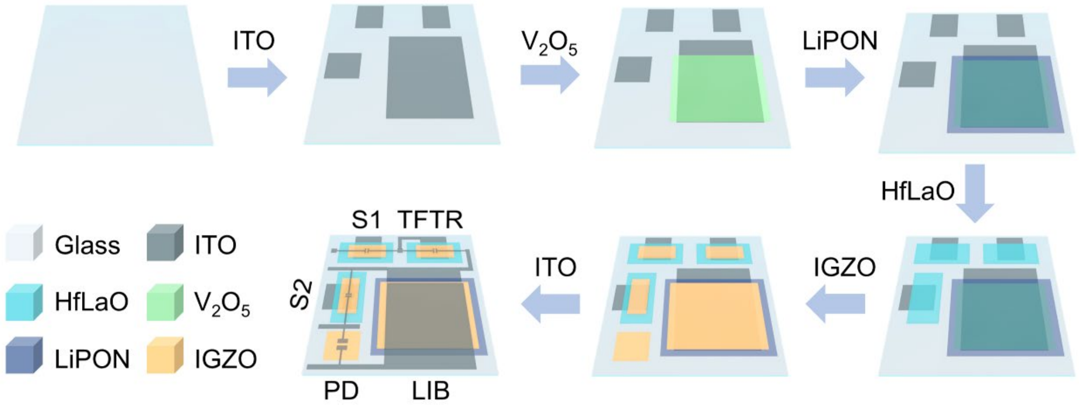

Supplementary Fig. 1. Fabrication processes of each component and the integrated transparent microsystem.

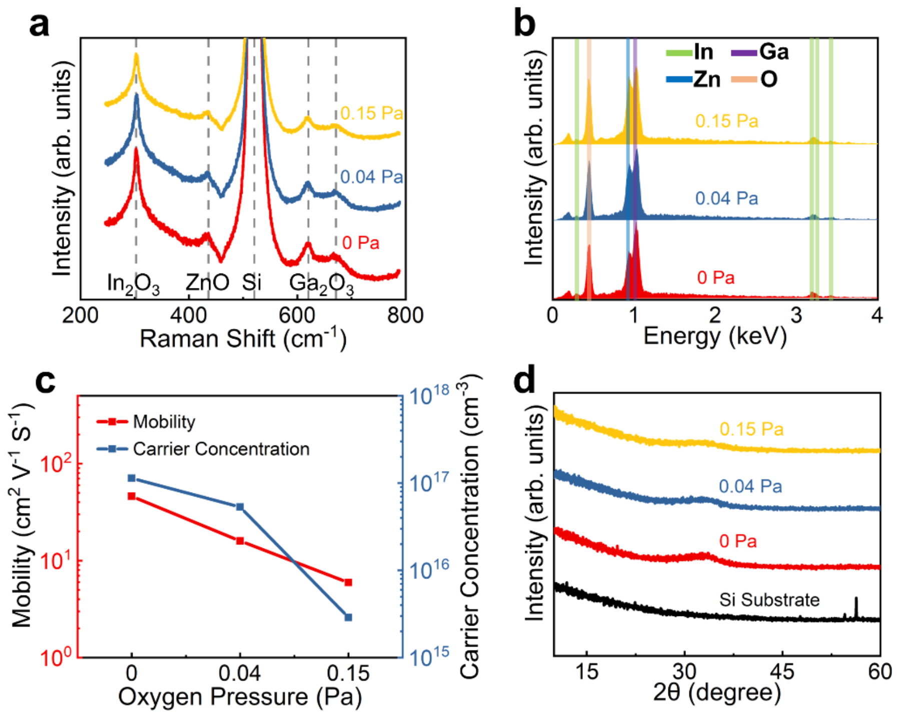

Supplementary Fig. 2. Material characterization of the IGZO films prepared at different P_{O2}. **(a)** Raman spectra. **(b)** EDS spectra. **(c)** Hall carrier concentration and mobility. **(d)** XRD patterns.

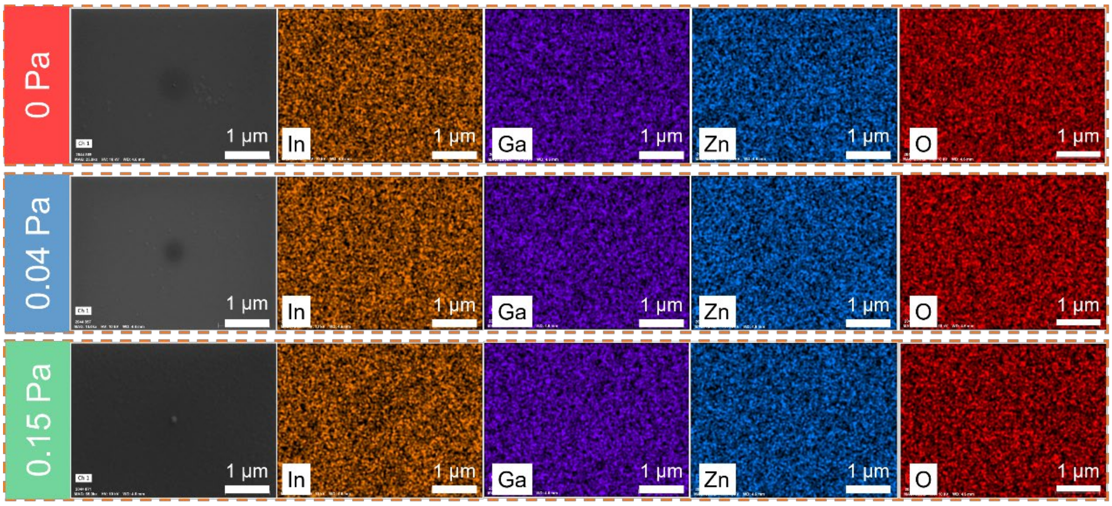

Supplementary Fig. 3. Plan-view SEM images and corresponding EDS elemental mappings of the IGZO films deposited at different P_{O2}.

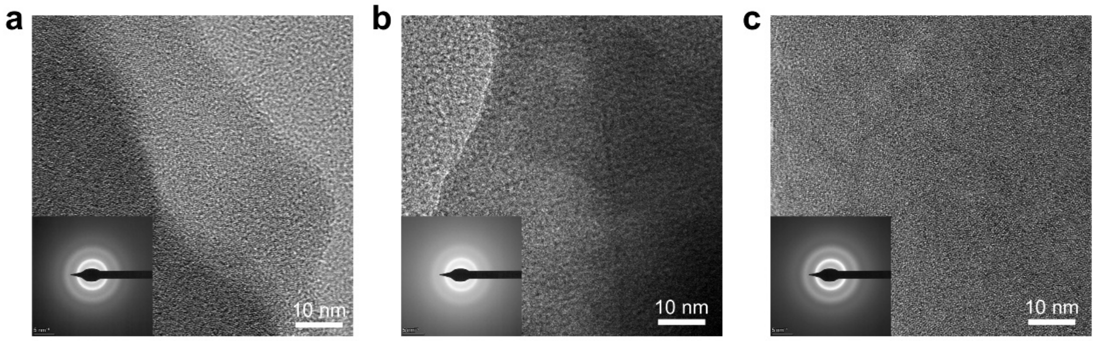

Supplementary Fig. 4. TEM images (inset is the SAED pattern) of the IGZO films deposited at **(a)** P_{O2} = 0 Pa, **(b)** P_{O2} = 0.04 Pa, and **(c)** P_{O2} = 0.15 Pa, respectively.

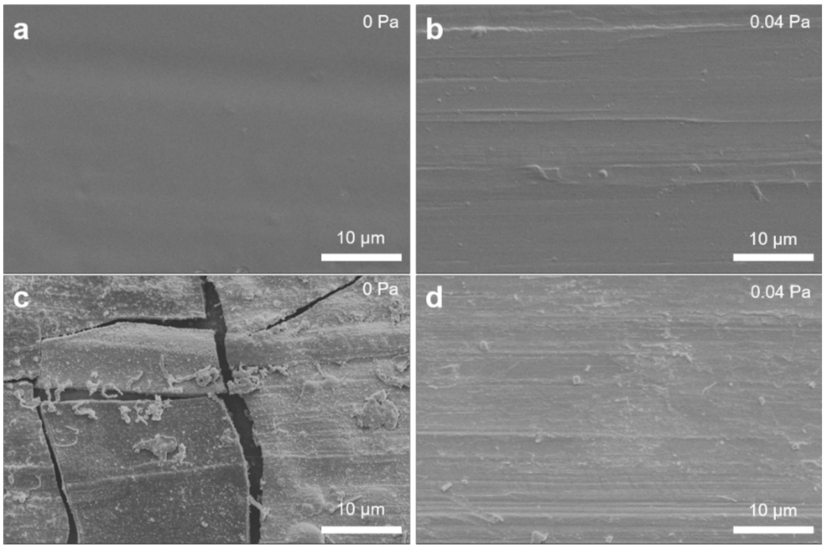

Supplementary Fig. 5. Plan-view SEM images of the IGZO anode films deposited at **(a)** P_{O2} = 0 Pa and **(b)** P_{O2} = 0.04 Pa before cycling. Plan-view SEM images of the IGZO anode films deposited at **(c)** P_{O2} = 0 Pa and **(d)** P_{O2} = 0.04 Pa after cycling.

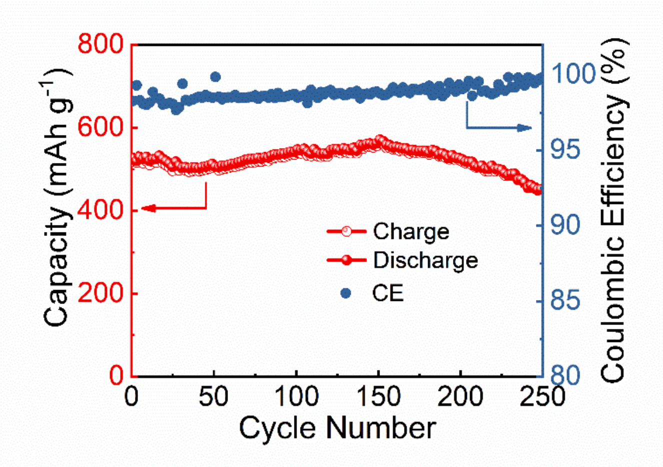

Supplementary Fig. 6. Cycling performance of the 800-nm IGZO anode film prepared at P_{O2} = 0.04 Pa based on the coin half cell.

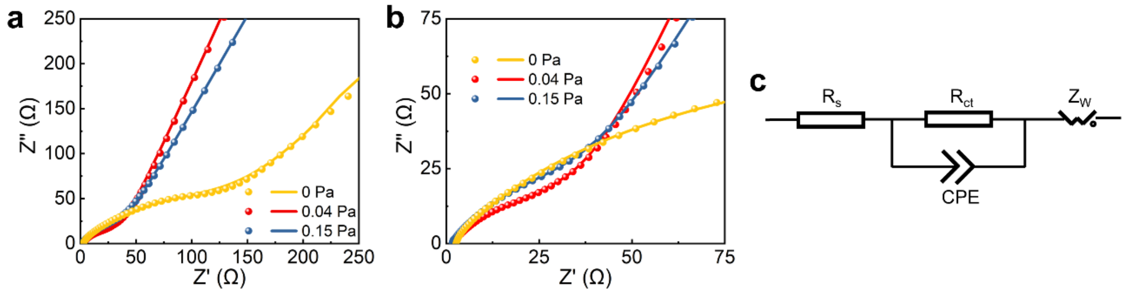

Supplementary Fig. 7. (a) EIS curves and **(b)** enlarged EIS curves as well as the fitting lines of the IGZO anode films prepared at different P_{O2}. **(c)** Corresponding equivalent circuit model. The raw data is represented in scatted dots, and the fitting results are represented in solid lines. The fitted data and the relative error between the raw and fitted data are shown in Supplementary Table 3.

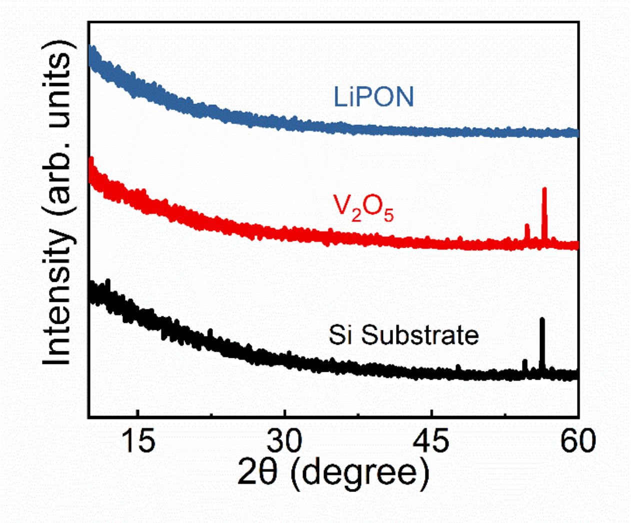

Supplementary Fig. 8. XRD diffraction patterns of the V_2O_5 and LiPON films deposited on the Si substrate.

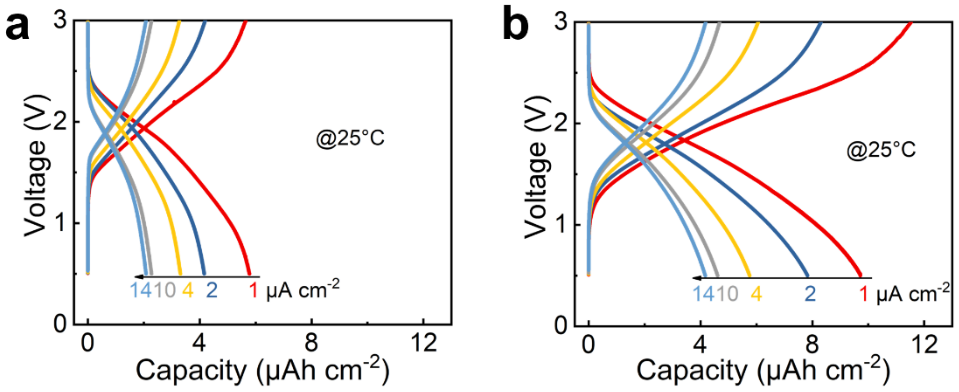

Supplementary Fig. 9. GCD curves under different current densities at 25 °C for the all-solid-state thin-film LIBs with an IGZO anode thickness of **(a)** 40 nm and **(b)** 120 nm, respectively. The LIBs are activated at a low current density of 1 μA cm^{-2} for three cycles firstly before testing.

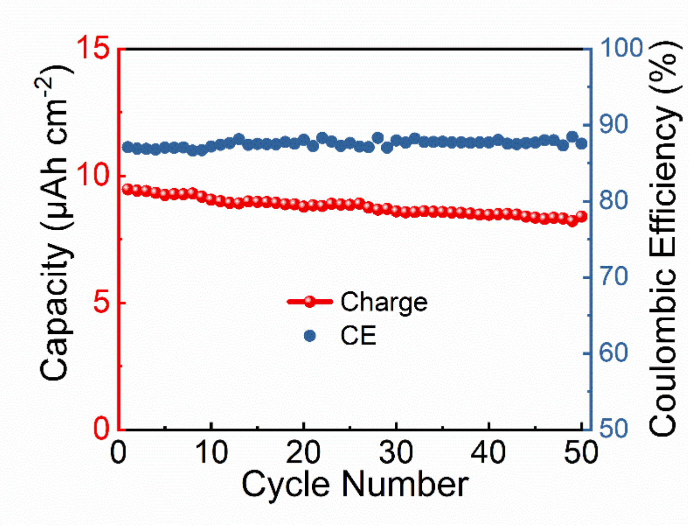

Supplementary Fig. 10. Cycling characteristics of the thin-film LIB at a current density of 14 μA cm^{-2} and 85 °C.

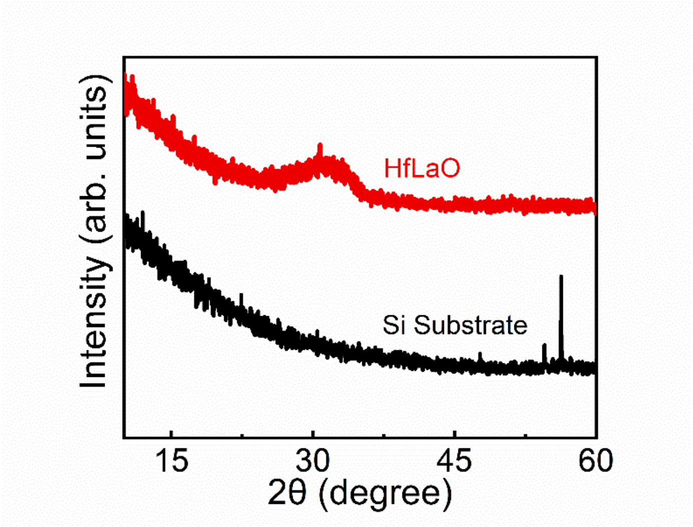

Supplementary Fig. 11. XRD diffraction patterns of the HfLaO gate dielectric film deposited on the Si substrate.

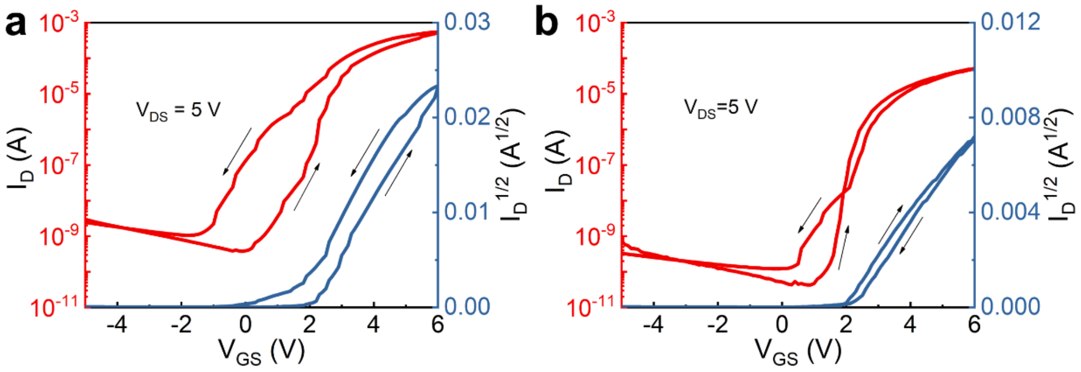

Supplementary Fig. 12. Transfer curves of the TFTs with IGZO channel layer prepared at **(a)** P_{O2} = 0 Pa and **(b)** P_{O2} = 0.15 Pa. The V_{th} is obtained from the intercept of $I_D^{1/2}$ vs. V_G in the saturation region with the *x*-axis. ΔV_{th} is defined by the threshold-voltage difference of the transfer curves under forward and backward sweepings.

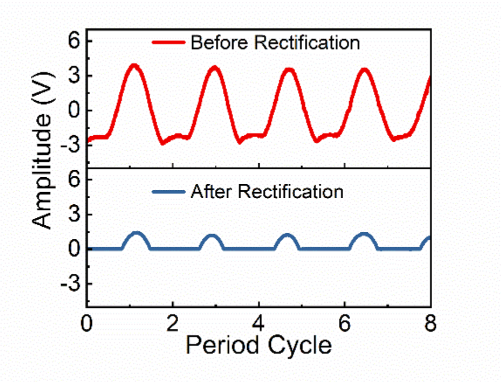

Supplementary Fig. 13. AC input signal generated by a homemade piezoelectric vibration energy harvester as well as the corresponding DC output signal processed by the TFTR rectifier.

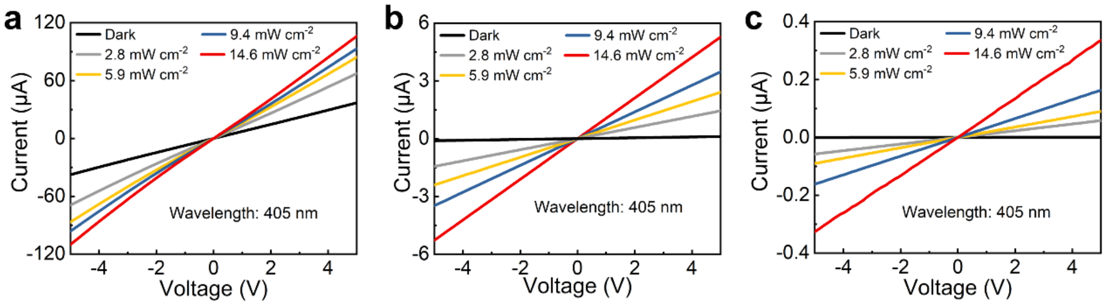

Supplementary Fig. 14. *I*−*V* curves of the PD devices under different power intensities at 405-nm light illumination and in the dark. **(a)** PD at P_{O2} = 0 Pa, **(b)** PD at P_{O2} = 0.04 Pa, and **(c)** PD at P_{O2} = 0.15 Pa.

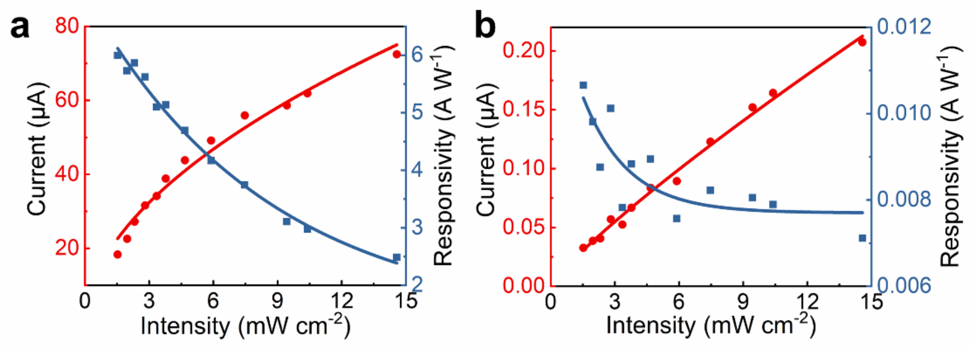

Supplementary Fig. 15. Dependence of photocurrent and responsivity on the 405-nm light intensity for the PD devices **(a)** at P_{O2} = 0 Pa and **(b)** at P_{O2} = 0.15 Pa.

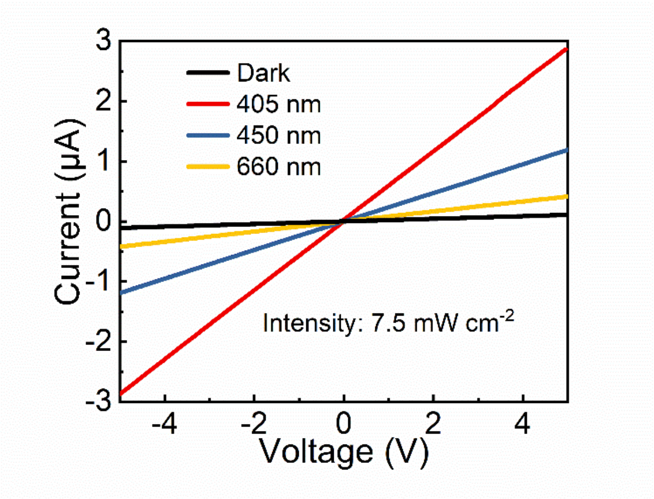

Supplementary Fig. 16. *I−V* curves of PD at P_{O2} = 0.04 Pa under different light wavelengths at 7.5 mW cm^{-2} and in the dark.

Supplementary Table 1. Deposition conditions of each layer for constructing the integrated transparent microsystem.

Film	Sputtering Target	Sputtering Power ($W\ cm^{-2}$)	Sputtering Pressure (Pa)	Sputtering Ambient (sccm)	Deposition Equipment
ITO	In_2O_3/SnO_2, 90/10 wt%	4.2	0.94	Ar = 40	Korvus
V_2O_5	V_2O_5	4.4	0.29	Ar/O_2 = 16/2	Korvus
LiPON	Li_3PO_4	6.4	0.59	N_2 = 13	Korvus
HfLaO	Target A: HfO_2 Target B: La	A: 2.1 B: 1.1	0.50	Ar/O_2 = 24/6	Sky
IGZO	$InGaZnO_4$	4.0	0.30	Ar/O_2 = 15/3	Sky
ITO	In_2O_3/SnO_2, 90/10 wt%	4.2	0.94	Ar = 40	Korvus

Supplementary Table 2. Relative atomic ratios of the In, Ga, Zn, and O elements in the IGZO films deposited at different P_{O2}. The atomic ratios are extracted based on the EDS spectra shown in Supplementary Fig. 2b.

Element / P_{O2}	In (%)	Ga (%)	Zn (%)	O (%)
0 Pa	16.12	20.92	11.78	51.19
0.04 Pa	16.06	19.20	12.88	51.86
0.15 Pa	16.20	17.90	13.74	52.15

Supplementary Table 3. Fitted data and relative error between the raw and fitted data obtained from Supplementary Fig. 7.

P_{O2}	0 Pa	0.04 Pa	0.15 Pa
R_S (error)	1.5 Ω (75.5%)	1.8 Ω (65.2%)	1.5 Ω (86.5%)
R_{CT} (error)	136.1 Ω (5.6%)	29.7 Ω (2.0%)	33.1 Ω (2.6%)
CPE-T (error)	1.0×10^{-4} (4.6%)	1.8×10^{-4} (4.1%)	5.9×10^{-5} (5.7%)
CPE-P (error)	0.6 (1.1%)	0.7 (0.7%)	0.7 (0.9%)
W-P (error)	0.3 (4.1%)	0.4 (0.6%)	0.3 (0.6%)

Supplementary Table 4. Performance comparison of the devices (including LIB, TFT and PD) prepared at various P_{O2}.

P_{O2}		0 Pa	0.04 Pa	0.15 Pa
**LIB**	Specific capacity (mAh g^{-1})	845.8	989.6	942.8
	Capacity retention (%)	67.1%	91.5%	94.8%
	Rate performance (mAh g^{-1})	17 (4 Ag^{-1})<br>274 (0.2A g^{-1})	227 (4 Ag^{-1})<br>616 (0.2A g^{-1})	149 (4 Ag^{-1})<br>310 (0.2A g^{-1})
**TFT**	Mobility (cm^2 V^{-1} s^{-1})	58.0	23.3	8.3
	Sub-threshold swing (mV dec^{-1})	458.9	209.2	180.0
	Threshold Voltage (V)	1.8	1.9	2.3
	Hysteresis (V)	0.6	0.4	0.3
	On-off current ratio	5.1×10^5	1.4×10^6	4.2×10^5
**PD**	Responsivity (A W^{-1})	6.0	0.35	0.01
	Light-to-dark current ratio	2.9	48.3	728.1

Supplementary Table 5. Performance comparison of the IGZO anode film at P_{O2} = 0.04 Pa in this work with other typical metal-oxide anode films in the literature.

**Anode materials**	**Reversible capacity (mAh g^{-1})**	**Initial CE (%)**	**Cycle number (capacity retention)**	**Rate performance (mAh g^{-1})**	**Ref.**
In_2O_3	195 @50μA cm^{-2}	10.0	10 (22.4%)	/	1
ZnO	435 @0.2A g^{-1}	56.1	100 (40.0%)	435 @0.2 A g^{-1}; 175 @3.2 A g^{-1}	2
Al_2O_3/ZnO	301 @125 mA g^{-1}	/	200 (49.8%)	/	3
TiO_2	327 @C/3	/	200 (77.6%)	/	4
TiO_2/SiO_2	560 @50μA cm^{-2}	56.6	100 (76.8%)	560 @50 μA cm^{-2}; 242 @1000 μA cm^{-2}	5
CuO/TiO_2	1036 @0.1C	60.5	10 (62.6%)	1036 @0.1C; 167 @2.0C	6
Cu_2O/TiO_2	520 @0.1C	53.6	10 (89.5%)	520 @0.1C; 370 @2.0C	6
Fe_2O_3	951 @100mA g^{-1}	71.0	200 (77.2%)	951 @100 mA g^{-1}; 510 @15 A g^{-1}	7
Fe_2O_3/Ag	1167 @0.1A g^{-1}	72.5	50 (83.5%)	1167 @0.1C; 509 @20.0C	8
LiPON/ $NiFe_2O_4$	917 @5μA cm^{-2}	67.7	50 (65.3%)	917 @5 μA cm^{-2}; 650 @25 μA cm^{-2}	9
NiO	1134 @ 0.1A g^{-1}	73.6	50 (45.5%)	1134 @0.1 A g^{-1}; 382 @0.8 A g^{-1}	10
InGaZnO	990 @50 mA g^{-1}	70.0	250 (89.2%)	616 @200 mA g^{-1}; 227 @4 A g^{-1}	This work

Supplementary Table 6. Performance comparison of the V_2O_5-based thin-film LIB in this work with those in the literature.

**Battery configuration**	**Specific capacity (μAh cm^{-2} μm^{-1})**	**Potential window (V)**	**Current density (μA cm^{-2})**	**Cycle number (capacity retention)**	**Ref.**
V_2O_5|LiPON|Li	33.3	1.5-3.8	30	80 (75%)	11
V_2O_5|LiPON|Li	32	2.15-3.8	64	1000 (93.8%)	12
$Li_{1.5}V_2O_5$|LiPON|Li	52	2.15-3.8	10	10 (96.2%)	13
V_2O_5-Li_3PO_4|LiPON|Li	16	0.5-4	2	30 (68.8%)	14
V_2O_5|LiPON|Li	54	2.15-3.8	10	60	15
V_2O_5-Li_3PO_4|LiPON|Li	38	0.5-3	0.6	30 (49%)	16
V_2O_5|LiPON|Li	20	2.15-3.8	10	50	17
LiV_2O5|Li_2PO_2N|SnN_x	40	0.5-3.3	50	100	18
V_2O_5|LiPON|IGZO	42.6	0.5-3.0	4.3	300 (96%)	This work

Supplementary Note 1

Calculation of the IGZO theoretic capacity

According to the literature, the theoretical capacity C_g (mAh g^{-1}) of the IGZO compositions (including In_2O_3, Ga_2O_3 and ZnO) can be calculated based on the following equations[19-21]

$$M_xO_y + 2yLi^+ + 2ye^- \Leftrightarrow yLi_2O + xM(M = In, \text{Ga}, Zn; x/y = 2/3, 2/3, 1/1\ for\ In, \text{Ga}, Zn) \quad (1)$$

$$M + zLi^+ + ze^- \Leftrightarrow Li_zM(z = 4.33, 2, 1\ f\text{or}\ In, Ga, Zn) \quad (2)$$

$$C_g = \frac{F\times n}{3.6\times W} \quad (3)$$

where F is the Faraday constant (96485 C mol^{-1}), W is the molecular weight and is equal to 277.6, 187.4 and 81.0 for In_2O_3, Ga_2O_3 and ZnO respectively, and n is the number of transferred electrons and can be calculated by

$$n = 2y + x \times z \quad (4)$$

n is determined to be 14.66, 10 and 3 for In_2O_3, Ga_2O_3 and ZnO, respectively.

Based on the above equations, the theoretic capacity for In_2O_3 (C_{In2O3}), Ga_2O_3 (C_{Ga2O3}) and ZnO (C_{ZnO}) is calculated to be 1415 mAh g^{-1}, 1430 mAh g^{-1} and 987 mAh g^{-1} respectively, which are consistent with those reported in the literature[19-21]

After that, the IGZO theoretic capacity (C_{IGZO}) can be calculated by

$$C_{IGZO} = C_{In_2O_3} \times m_{In_2O_3} + C_{Ga_2O_3} \times m_{Ga_2O_3} + C_{ZnO} \times m_{ZnO} \quad (5)$$

where m_{In2O3}, m_{Ga2O3} and m_{ZnO} represent mass ratio of In_2O_3, Ga_2O_3 and ZnO in the IGZO film ($InGaZnO_4$ according to the target used in this work), and are equal to 0.443, 0.299 and 0.258 respectively. Consequently, C_{IGZO} is determined to be 1311 mAh g^{-1}. The experimental capacity of the IGZO film is a little smaller than the theoretic value. The electrochemical reactions displayed in equations (1) and (2) would enhance by decreasing the charge/discharge current, thus helping make the experimental capacity closer to the theoretic value.

Supplementary References

1. Ho, W. H., Li, C. F., Liu, H. C. & Yen, S. K. Electrochemical performance of In_2O_3 thin film electrode in lithium cell. *J. Power Sources* **175**, 897-902 (2008).

2. Yuan, J. et al. Facile fabrication of three-dimensional porous ZnO thin films on Ni foams for lithium ion battery anodes. *Mater. Lett.* **190**, 37-39 (2017).

3. Shi, Q. et al. Electrochemical and optoelectric behavior of Al-doped ZnO films as transparent anode for Li-ion batteries. *Mater. Today Commun.* **19**, 471-475 (2019).

4. Nagpure, S. et al. Layer-by-layer synthesis of thick mesoporous TiO_2 films with vertically oriented accessible nanopores and their application for lithium-ion battery negative electrodes. *Adv. Funct. Mater.* **28**, 1801849 (2018).

5. Wu, J. et al. Rapid construction of TiO_2/SiO_2 composite film on Ti foil as lithium-ion battery anode by plasma discharge in solution. *Appl. Phys. Lett.* **114**, 043903 (2019).

6. Barreca, D. et al. On the performances of Cu_xO-TiO_2 (x = 1, 2) nanomaterials as innovative anodes for thin film lithium batteries. *ACS Appl. Mater. Interfaces* **4**, 3610-3619 (2012).

7. Teng, X. et al. A nanocrystalline Fe_2O_3 film anode prepared by pulsed laser deposition for lithium-ion batteries. *Nanoscale Res. Lett.* **13**, 60 (2018).

8. Zhang, D., Li, Y., Yan, M. & Jiang, Y. Fe_2O_3-Ag porous film anodes for ultrahigh-rate lithium-ion batteries. *ChemElectroChem* **1**, 1155-1160 (2014).

9. Wei, K. et al. Lithium phosphorous oxynitride (LiPON) coated $NiFe_2O_4$ anode material with enhanced electrochemical performance for lithium ion batteries. *J. Alloys Compd.* **769**, 110-119 (2018).

10. Cao, L., Wang, D. & Wang, R. NiO thin films grown directly on Cu foils by pulsed laser deposition as anode materials for lithium ion batteries. *Mater. Lett.* **132**, 357-360 (2014).

11. Oukassi, S. et al. Ultra-thin rechargeable lithium ion batteries on flexible polymer: design, low temperature fabrication and characterization. *J. Electrochem. Soc.* **164**,

A1785-A1791 (2017).

12. Xiao, C. F. et al. Ensemble design of electrode-electrolyte interfaces: toward high-performance thin-film all-solid-state Li-metal batteries. *ACS Nano* **15**, 4561-4575 (2021).

13. Navone, C. et al. Lithiated c-V_2O_5 thin-film as positive electrode for rocking-chair solid-state lithium microbattery. *Ionics* **16**, 577-580 (2010).

14. Tsuji, K., Yoshida, M. & Kanno, I. Fabrication of all-solid-state amorphous thin-film Lithium-ion batteries. *PowerMEMS 2021 Virtual Conference* 216-219 (2021).

15. Navone, C., Baddour-Hadjean, R., Pereira-Ramos, J. P. & Salot, R. Sputtered crystalline V_2O_5 thin films for all-solid-state lithium microbatteries. *J. Electrochem. Soc.* **156**, A763-A767 (2009).

16. Kanazawa, S. et al. Deposition and performance of all solid-state thin-film lithium-ion batteries composed of amorphous Si/LiPON/VO-LiPO multilayers. *Thin Solid Films* **697**, 137840 (2020).

17. Oukassi, S., Salot, R. & Pereira-Ramos, J. P. Elaboration and characterization of crystalline RF-deposited V_2O_5 positive electrode for thin film batteries. *Appl. Surf. Sci.* **256**, 149-155 (2009).

18. Pearse, A. et al. Three-dimensional solid-state lithium-ion batteries fabricated by conformal vapor-phase chemistry. *ACS Nano* **12**, 4286-4294 (2018).

19. Ho, W. H., Li, C. F., Liu, H. C. & Yen, S. K. Electrochemical performance of In_2O_3 thin film electrode in lithium cell. *J. Power Sources* **175**, 897-902 (2008).

20. Guo, J. et al. Novel strategy of constructing hollow Ga_2O_3@N-CQDs as a self-healing anode material for lithium-ion batteries. *ACS Sustainable Chem. Eng.* **8**, 13692-13700 (2020).

21. Cao, Y. Q., Wang, S. S., Liu, C., Wu, D. & Li, A. D. Atomic layer deposition of ZnO/TiO2 nanolaminates as ultra-long life anode material for lithium-ion batteries. *Sci. Rep.* **9**, 11526 (2019).
